# A deep learning pipeline for detecting vestibular schwannoma patients with unilateral vestibular loss based on kinematic data

**DOI:** 10.1038/s41598-025-29776-8

**Published:** 2025-11-25

**Authors:** Lou C. Kohler Voinov, Sergio Sanchez-Manso, Raabeae Aryan, Jennifer L. Millar, Michael C. Schubert, Kathleen E. Cullen

**Affiliations:** 1 Department of Biomedical Engineering, Tsinghua University, Beijing, 100084 China; 2 Department of Biomedical Engineering, Johns Hopkins University School of Medicine, Baltimore, MD 21205 USA; 3 Department of Physical Medicine and Rehabilitation, Johns Hopkins University School of Medicine, Baltimore, MD USA; 4 Department of Otolaryngology-Head and Neck Surgery, Johns Hopkins University School of Medicine, Baltimore, MD 21205 USA; 5 Department of Neuroscience, Johns Hopkins University School of Medicine, Baltimore, MD 21205 USA; 6 Kavli Neuroscience Discovery Institute, Johns Hopkins University, Baltimore, MD 21218 USA

**Keywords:** Vestibular schwannoma, Functional gait assessment, Kinematics, Machine learning, Deep learning, Convolutional neural network, Health care, Medical research, Neurology, Neuroscience

## Abstract

Detecting subtle balance and coordination impairments in individuals with vestibular schwannomas (VS) remains a challenge, particularly when traditional clinical assessments appear normal. Focusing on patients prior to surgical intervention, this study offers a unique opportunity to capture early motor adaptations before permanent vestibular loss. We developed a deep learning-based classifier to distinguish VS patients from age-matched healthy controls using kinematic data collected during standardized gait tasks. Participants performed a short-duration (< 10 s) straight-path walk at a normal pace (an item from the Functional Gait Assessment) and a 30-second walk with intermittent eye closure. Six inertial measurement units (IMUs) were placed at different body locations, and models were trained using data from each sensor independently. We employed a convolutional neural network (CNN) tailored to this clinical application. The classifier achieved a maximum accuracy of 0.74 for controls and 0.71 for VS patients. Notably, the model detected early-stage compensatory movement patterns—even in the absence of clinical score differences—by extracting features from wrist and trunk sensors, regions not emphasized in standard evaluations. Performance improved with dataset size, particularly with more subjects, highlighting the value of broader data collection in clinical machine learning. Pretraining on external datasets that included both healthy and pathological subjects further improved accuracy, underscoring the importance of dataset diversity. These findings demonstrate the potential of deep learning to detect functional impairment when clinical scoring falls short and provide practical guidance for sensor placement, dataset design, and model training in vestibular and balance disorders.

**Supplementary Information:**

The online version contains supplementary material available at 10.1038/s41598-025-29776-8.

## Introduction

 Vestibular schwannomas (VS) are benign tumors arising from the eighth cranial nerve, accounting for approximately 6–7% of all intracranial tumors^1,2^. These tumors often lead to hearing loss, facial nerve dysfunction, tinnitus, vertigo, and, critically, impairments in balance and coordination. VS frequently disrupt gaze and gait stability during standing or walking, resulting in dizziness, increased risk of falling^3,4^, and movement abnormalities that can distinguish patients from healthy individuals^5,6^. To assess functional deficits in VS patients, the current standard of care involves the use of the Functional Gait Assessment scale (FGA), a standardized battery of tasks to evaluate postural stability during gait^7^. The full FGA includes the following tasks: (1) gait on a level surface, (2) change in gait speed, (3) gait with horizontal head turns, (4) gait with vertical head turns, (5) gait and pivot turn, (6) step over obstacle, (7) gait with narrow base of support, (8) gait with eyes closed, (9) ambulating backwards, and (10) stair negotiation. However, the FGA is subjectively scored by clinicians and relies on a coarse integer scale (0–3), potentially overlooking specific kinematic abnormalities that can be quantified over time and scored on a continuum^7,8^. As a result, critical yet subtle movement differences—especially those outside the clinicians’ immediate focus—may remain undetected^5,6^. This limitation is particularly relevant for patients evaluated prior to surgery, when vestibular input is impaired but not yet permanently lost, and early motor adaptations may already be present. Identifying functional impairments during this pre-surgical window may enable earlier diagnosis, inform treatment planning, and support targeted rehabilitation.

Wearable inertial measurement units (IMUs) offer a promising alternative to subjective clinical scoring by enabling continuous, objective quantification of movement patterns during everyday activities. These sensors are inexpensive, non-invasive, portable, and capable of capturing full-body 3D kinematics by measuring acceleration and angular velocity^9^. Prior work has demonstrated that IMUs can be used to accurately detect gait cycles and compute spatiotemporal metrics (e.g., stride length, swing time, gait speed, head and body kinematics)^8,9^. In the context of vestibular disorders, quantification of IMU data during a variety of standardized tests^10–12^ has proven to be effective in distinguishing between patient and control kinematics^5,6,13,14^. Prior studies have largely focused on head-mounted sensors because head movements directly stimulate the vestibular system, which is located in the inner ear and responsible for detecting self-motion^6,13–15^. In contrast, less attention has been paid to other body segments, even though they may reveal important movement patterns. This is especially relevant during tasks like the FGA, where subtle limb-specific impairments can be difficult for clinicians to observe and interpret visually.

The use of machine learning (ML) offers a powerful approach to analyzing high-dimensional IMU data without manual feature selection or the loss of original signal integrity. By learning mappings from input signals to diagnostic labels, ML models enable the extraction of relevant features without expert domain knowledge. Within the field of clinical vestibular research, machine learning (ML) has primarily been applied to the analysis of static or clinical data, such as electronic health records, videonystagmography, or posturography^16–21‚61^. Prior studies have used wearable kinematic sensors in combination with machine learning to classify vestibular pathologies and pathologies with vestibular related symptoms. These investigations^20,22–28^ demonstrated that IMUs combined with ML algorithms could be used to distinguish patients with vestibular symptoms from healthy controls. However, these cohorts included a heterogeneous set of diagnoses rather than a specific condition such as vestibular schwannoma (VS). As a result, it remains unclear whether kinematic data can reliably differentiate VS patients from controls. Furthermore, previous studies have not systematically examined how sensor placement, dataset size, or pretraining strategies impact classification accuracy. To address these limitations, we developed a deep learning framework tailored to wearable sensor data in a well-defined VS population, with a focus on detecting subclinical motor adaptations. Our work is motivated by the increasing use of IMUs both in the clinic and rehabilitation settings, where sensor placement varies widely across studies^29–33^. In the field of algorithm development using kinematic data, the impact of sensor placement on model performance is also being actively investigated^34–40^. The present study bridges these two areas by systematically investigating how sensor location influences the performance of our clinically targeted algorithm.

We leveraged full-body IMU data collected from VS patients prior to surgical intervention to train a deep learning model that distinguishes them from age-matched healthy controls. Kinematic signals were recorded from six body locations during two gait tasks—a standard short walk and a longer-duration walk with intermittent eye closure. Recognizing the challenges of limited sample sizes in clinical settings, we also evaluated strategies to enhance performance in low-data regimes, including optimal sensor placement and pretraining on external datasets. Our goals were twofold: (1) to develop a kinematics-based classifier that may serve as a digital biomarker for VS monitoring, and (2) to offer practical guidance for applying ML to wearable sensor data in vestibular populations. Importantly, our model detected differences even during minimally challenging tasks, underscoring its sensitivity to early-stage compensatory strategies. By focusing on patients under pre-operative surveillance, our findings demonstrate the potential of wearable-based classification to support diagnosis and track functional decline over time.

## Methods

### Dataset

The dataset used in this study consisted of kinematic recordings of gait from 32 individuals diagnosed with vestibular schwannoma (VS) and 32 age-matched healthy controls, collected between 2019 and 2021 at a sampling rate of 500 Hz. Patients had come to the Johns Hopkins clinic with symptoms including hearing loss, ear fullness, tinnitus, imbalance, dizziness, facial pain, and numbness. 3 T MRI imaging was used to confirm the presence of the tumor (either a Philips or Siemens MRI scanner). Tumor volume ranged from 0.03 cm^3^ to 21.07 cm^3^ (average = 3.34, standard deviation = 5.06). VS subjects all had unilateral lesions—13 left-sided and 19 right-sided—and had not undergone any surgical intervention for their tumor at the time of data collection. Healthy controls were selected using an age-matching protocol (± 10 years). Eligibility included no history of otologic (other than VS) or neurological disease in both groups. Participant ages ranged from 25 to 81 years, with 45.3% male (29/64) and 54.7% female (35/64). Additional demographic details are provided in Table 1.

**Table 1:** Dataset gender and age information.

Gender	Parameter	Control	VS
All	Count	32	32
	Mean Age ± SD (years)	55.5 *±* 17.6	57.4 *±* 11.4
	Age Range	25–74	26–81
Male	Count	14	15
	Mean Age ± SD (years)	58.8 *±* 16.2	56.9 *±* 10.8
	Age Range	28–74	39–72
Female	Count	18	17
	Mean Age ± SD (years)	52.9 *±* 18.2	57.4 *±* 11.4
	Age Range	25–69	26–81

VS: vestibular schwannoma. SD: standard deviation.

Participants performed two tasks: (1) a < 10-second straight path walk at a comfortable pace, and (2) a longer 30-second walk with slow blinks (i.e., eye closure for two steps followed by eye opening for one step, repeated intermittently). The shorter walk on level ground corresponds to Task 1 of the 10-item Functional Gait Assessment (FGA), a standardized clinical tool used to evaluate postural stability during walking across diverse conditions. The maximum FGA score is 30, with higher scores indicating better gait function. All participants in this study completed the full FGA, and clinical scores were recorded.

For the kinematic analysis, only data from Task 1of the FGA and the extended blinking task were used. These two exercises were selected as the initial focus of this preliminary study due to their relative simplicity compared to other FGA gait tasks. Notably, FGA Task 1 (gait on a level surface) is considered one of the least challenging tasks in the battery, since it requires only straight-path walking at a self-selected pace without additional cognitive or sensorimotor demands. Future studies should expand upon this foundation by incorporating additional FGA tasks to comprehensively evaluate model generalizability. All 64 subjects completed the shorter task; however, due to unusable recordings from one subject in each group, the longer task included 31 subjects per group (62 total). Task-specific sample counts are provided in Table 2. This prospective study was approved by the Johns Hopkins Institutional Review Board and conducted in accordance with institutional guidelines for ethical human subject research. Written informed consent was obtained from all participants.

**Table 2:** Distribution of subjects and samples by task. Similar proportions are present between the number of samples per label. A sample is defined as one gait cycle from one subject.

Task	Type	#subjects	#samples	% of samples
Walking on level ground (*<* 10 s)	All	64	312	100.00
	Control	32	153	49.04
	VS	32	159	50.96
Walking with slow blinks (30s)	All	62	1393	100.00
	Control	31	690	49.53
	VS	31	703	50.47

## Gait segmentation and preprocessing

**Fig. 1:**
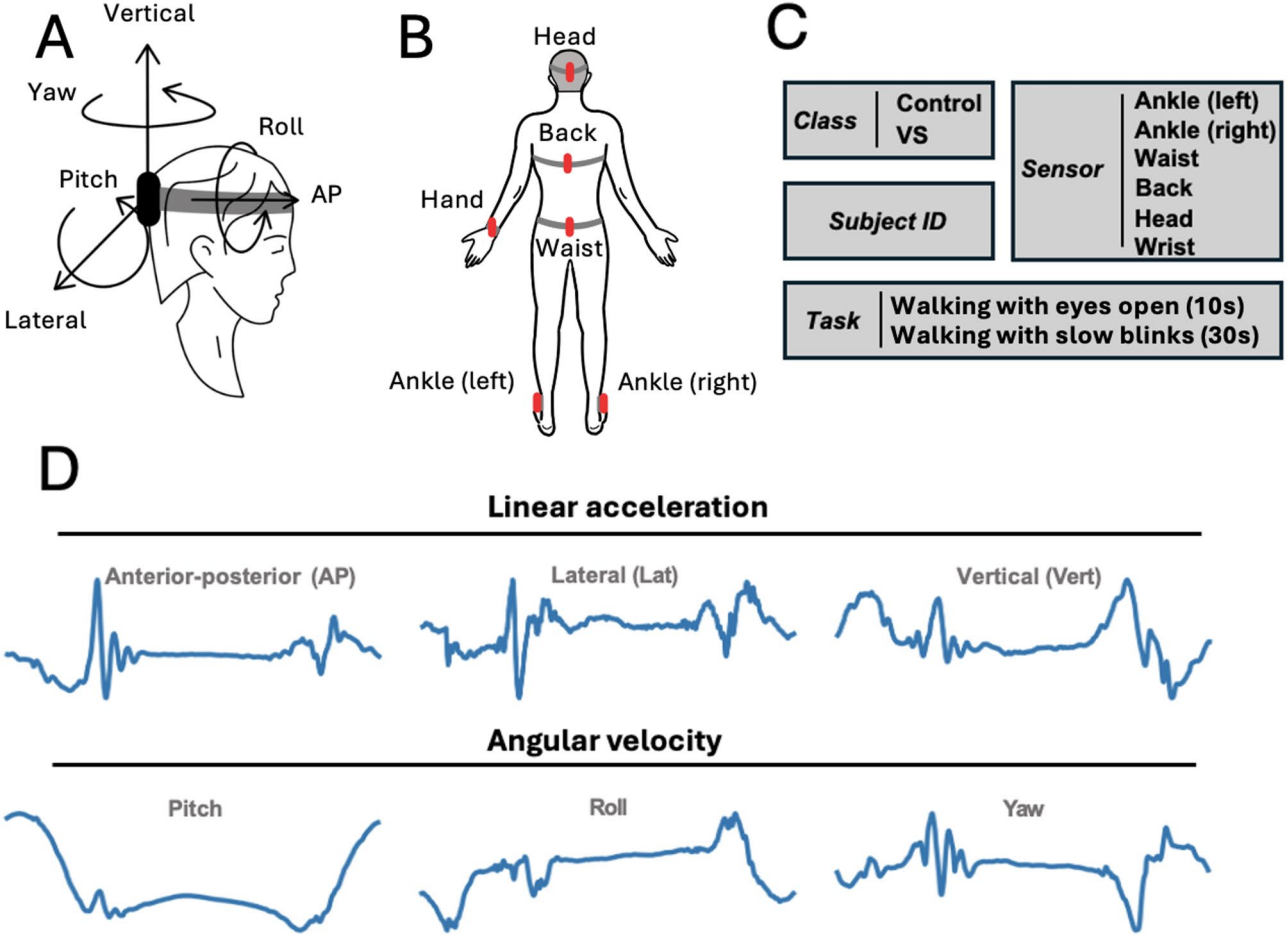
General descriptors of the data used throughout the experiment. (**A**) Axes of recorded dimensions. (**B**) Sensor placements. The wrist sensor is placed on the dominant side of the subject. (**C**) Possible values of the labels incorporated in any given gait cycle (sample) besides the kinematic data. (**D**) Components of the kinematic information of a given gait cycle (sample), forming a 6 × 512 matrix. Data from a healthy subject performing walking with eyes open task.

All participants completed the walking tasks while wearing six inertial measurement units (IMUs; Shimmer3, Shimmer Research, Dublin, Ireland; 51 × 34 × 14 mm^3^). Sensors were placed on the dominant wrist (distal of dominant forearm immediately above and dorsal of the wrist joint), each ankle (distal-lateral aspect of both shanks immediately above the lateral malleoli), the waist (approximately between L4-S1 vertebrae), the upper back (back of upper trunk at the level of T6-T7 vertebrae), and the back of the head. Each IMU recorded 3D linear acceleration (anterior-posterior, mediolateral, vertical) and 3D angular velocity (pitch, roll, yaw) at each time point, yielding six data channels per sensor. The raw recordings included the entire duration of both walking task. Each IMU was calibrated using the manufacturer’s default parameters. Because each IMU records data within its own local coordinate frame, rotation matrices were applied to transform all IMUs into a common external global coordinate frame. Data were segmented into individual gait cycles using the pitch angular velocity signal from the right ankle IMU, following methods described in Zobeiri et al.^**41**^ Each cycle was then resampled to a fixed length of 512 time points, effectively down sampling by selecting linearly spaced points, using linear interpolation. This resulted in a consistent 6 × 512 input matrix per sample—6 signal dimensions (from one sensor) across 512 time steps. No additional filtering, apart from down sampling, was applied to the raw IMU signals. Figure 1 illustrates the data structure and segmentation pipeline.

## Machine learning model

To classify kinematic signals, we implemented a convolutional neural network (CNN) architecture designed for time-series data and having shown promise in past work classifying human gait signals^42–45^. All analyses were conducted in Python, and the model was constructed de novo in Pytorch. The model consisted of two primary stages: a convolutional stage that processes each signal dimension independently, followed by a dense classification stage. Specifically, each input dimension was passed through its own independent set of 1-D convolutions, a method which has been described in past work^45–47^. The outputs from all six dimensions were then concatenated at the final dense layer. Dropout and batch normalization layers were incorporated to enhance regularization and overall model performance. In particular, the batch normalization mitigated inter-individual signal variation that could otherwise bias model outputs. The model’s convolutional stage consists of six parallel branches—one per input dimension—each created using four repetitions of a 1-D convolutional layer, rectified linear unit (ReLU) activation function layer, a batch normalization layer, and max pooling layer.

**Fig. 2:**
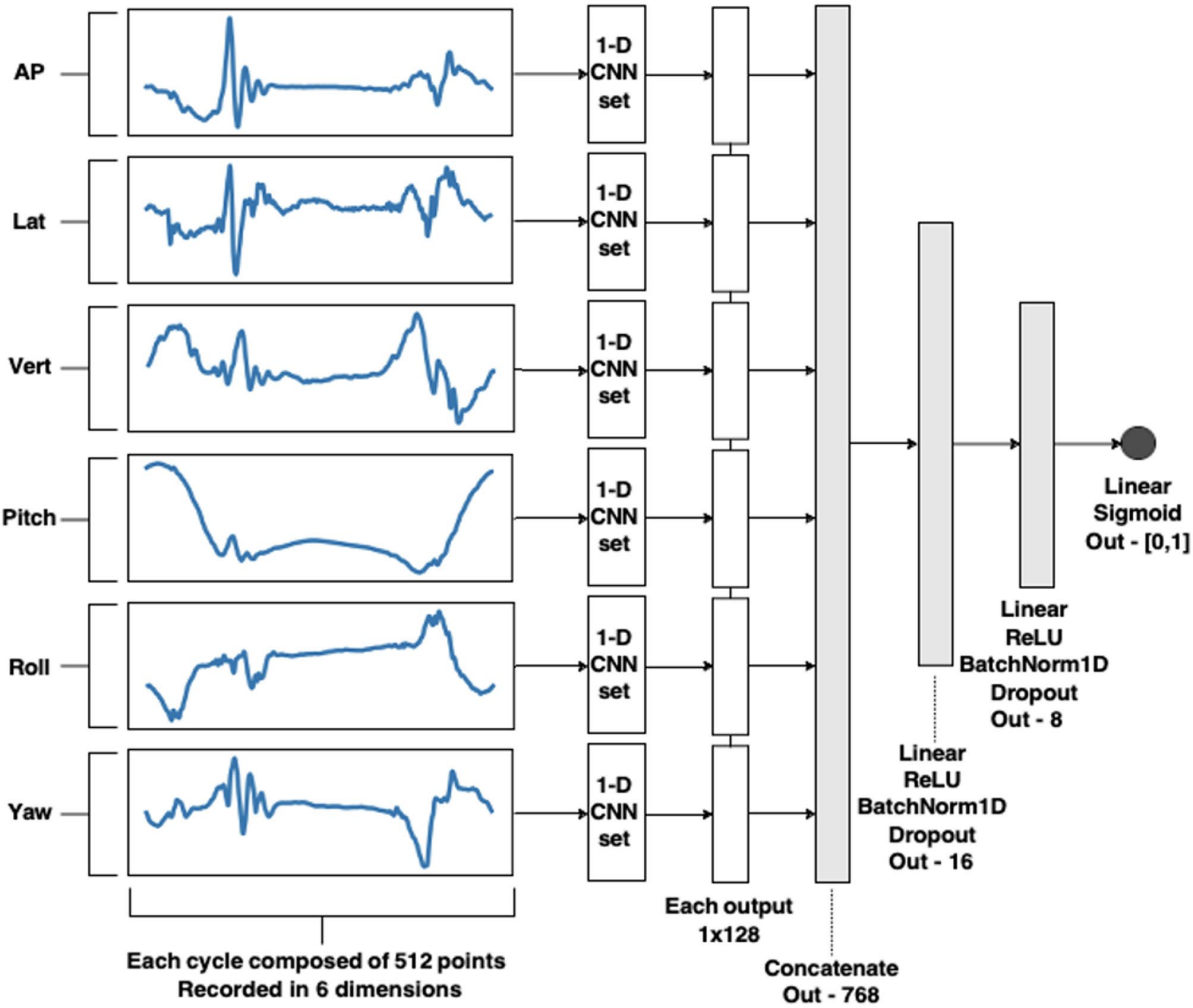
Diagram of the overview of the ML model. All *Linear* layers use the previous layer size as #input features, and the current layer size as #output features. All *BatchNorm1D* use the current layer size as the number of features. All *Dropout* rates were 50%.

**Fig. 3:**
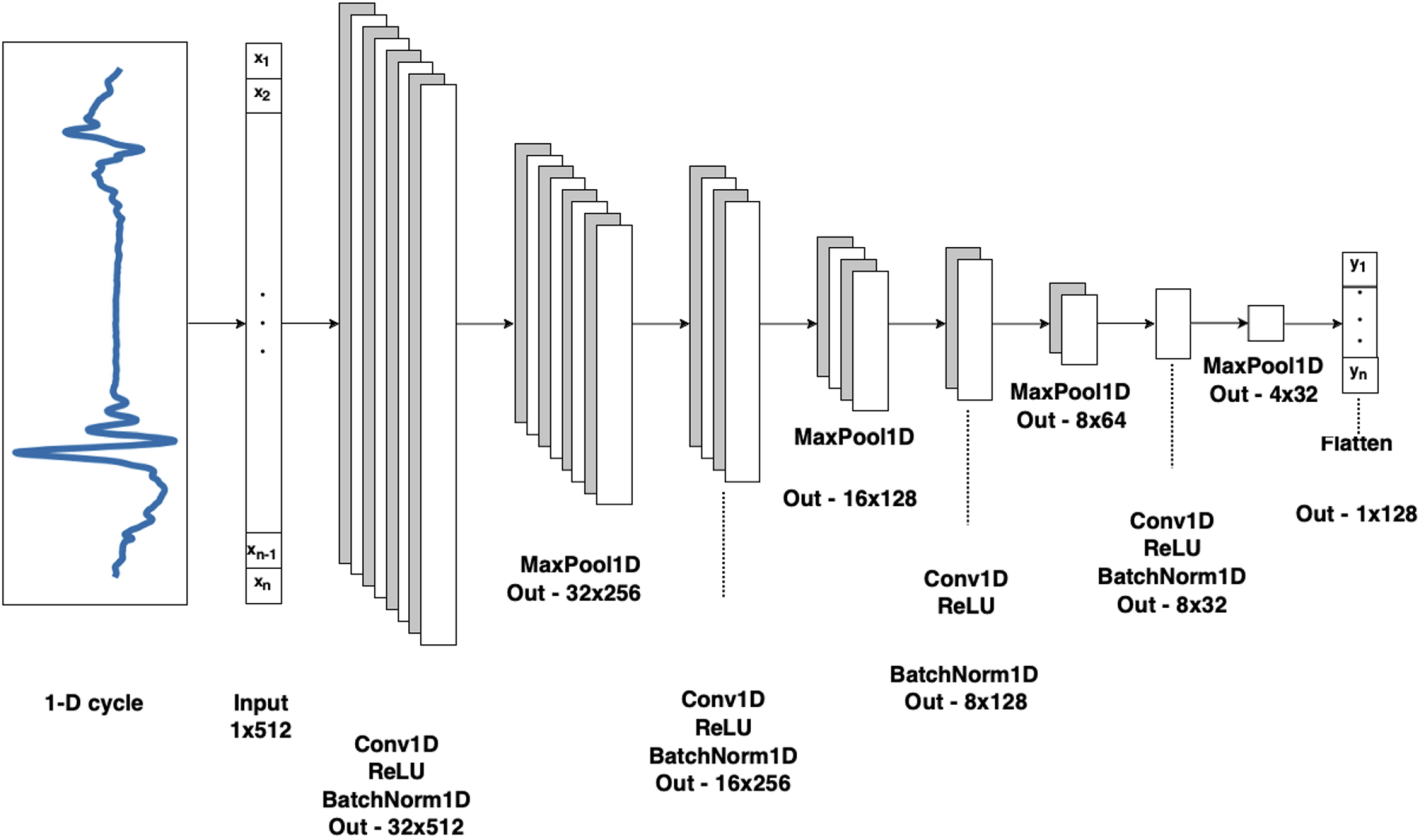
Zoomed-in diagram of the repeated parallel 1-D CNN set for each dimension. All 2-D data sizes are noted as [#filters]x[#time points]. All *Conv1D* layers use the previous layer #filters as #input channels, the current layer #filters as #output channels, a kernel size of 3, a padding of 1, and a stride of (1) All *MaxPool1D* use a kernel size of 2, and a stride of (2) All *BatchNorm1D* use the current layer #filters as number of features.

After processing with each parallel convolutional set, the output of each branch is flattened and concatenated into a single feature vector, which is passed to the dense stage. This dense stage comprised two fully connected layers, each followed by ReLU activation, batch normalization, and dropout layers. A final fully connected layer with a single output neuron performed the binary classification, with the output passed through a sigmoid activation function and a threshold of 0.5 to determine class membership. Specific kernel sizes and output dimensions for both the convolutional and dense stages are detailed in Figs. 2 and 3. The model was trained for 100 epochs using a batch size of 64, a learning rate of 0.1, and a step-based learning rate scheduler (step size = 25, γ (decay rate) = 0.1). Binary cross-entropy was used as the loss function, and the Adam optimizer^48^ was employed for training. A table recapitulating all parameters can be found in Supp Table 7.

## Training and evaluation protocol

**Fig. 4:**
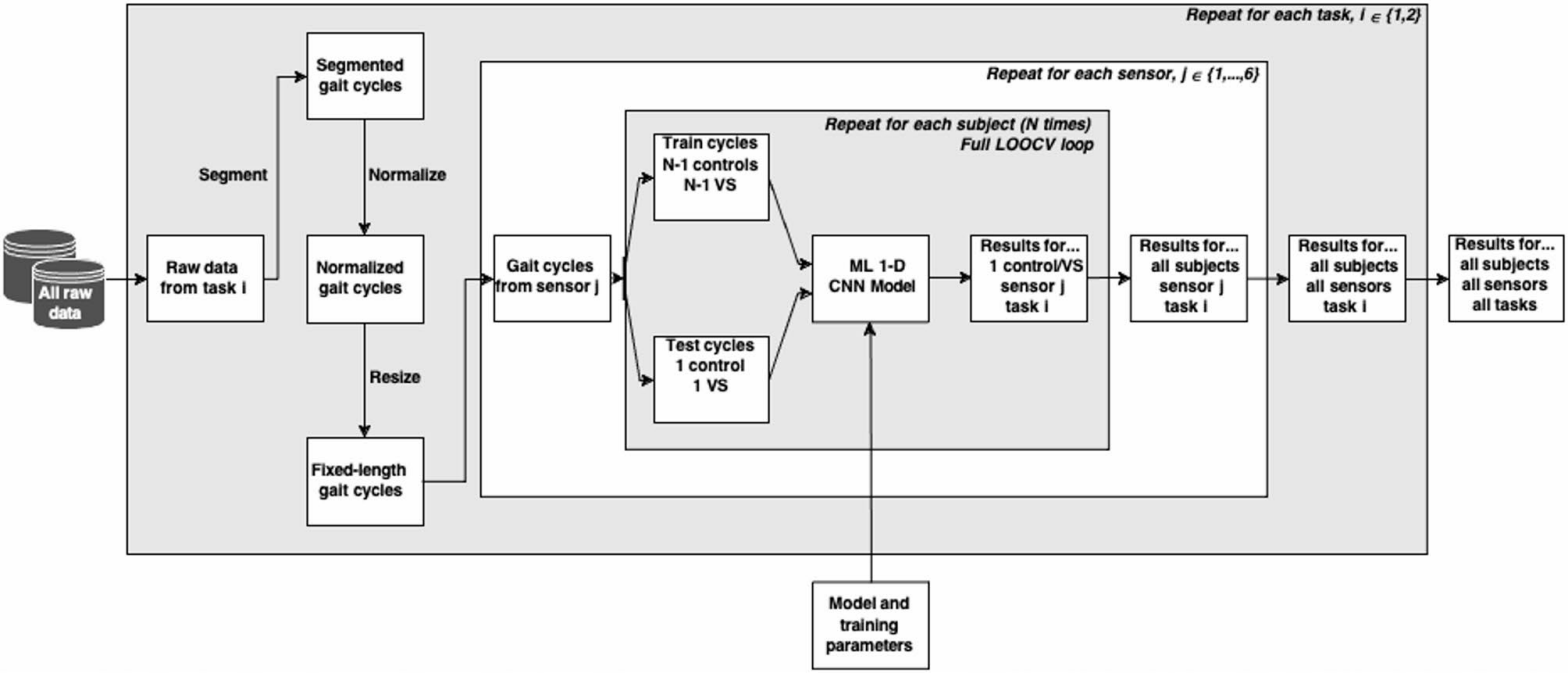
Diagram of the overall pipeline used throughout the paper. The order of separation of data goes first by tasks, then by sensor, and lastly by subjects. Note that the process of segmentation, normalization, and resizing is done for each task but before dividing the data into sensors. The N value that determines the number of iterations on the LOOCV loop will depend on the subjects present at the task of study, regardless of the sensor. The model and training parameters remain constant for all tasks, sensors, and subjects. The results for 1 subject, 1 sensor, and 1 task include the average rate of classification (between control/VS) success for all of the samples (gait cycles) of that subject.

To prevent data leakage, the train-test split was performed at the subject level rather than by individual cycles. All input data were normalized based on the value range of the training set. Model evaluation was conducted using Leave-One-Out Cross-Validation (LOOCV), where each iteration held out one control subject and one VS subject for testing, while the remaining subjects formed the training set. This approach maximized inter-subject variability during training. For each iteration, the model’s performance was assessed by computing the accuracy across all gait cycles for each test subject. Final accuracy is reported as the average of these per-subject means, separately for the control and VS groups, using a scale from 0 to 1^49^ (see Eq. 1). Accordingly, for each sensor-task combination, the model was trained and evaluated N times, where N equals the number of subjects in either group (i.e., half the dataset). This entire process was repeated independently for each sensor input. A schematic of the full pipeline is provided in Fig. 4. All training and testing were conducted using an NVIDIA A100 PCIe 40GB GPU.

1 $$\:\begin{array}{c}Accuracy=\:\frac{\boldsymbol{T}\boldsymbol{r}\boldsymbol{u}\boldsymbol{e}\:\boldsymbol{p}\boldsymbol{o}\boldsymbol{s}\boldsymbol{i}\boldsymbol{t}\boldsymbol{i}\boldsymbol{v}\boldsymbol{e}+\boldsymbol{T}\boldsymbol{r}\boldsymbol{u}\boldsymbol{e}\:\boldsymbol{n}\boldsymbol{e}\boldsymbol{g}\boldsymbol{a}\boldsymbol{t}\boldsymbol{i}\boldsymbol{v}\boldsymbol{e}}{\boldsymbol{T}\boldsymbol{r}\boldsymbol{u}\boldsymbol{e}\:\boldsymbol{p}\boldsymbol{o}\boldsymbol{s}\boldsymbol{i}\boldsymbol{t}\boldsymbol{i}\boldsymbol{v}\boldsymbol{e}+\boldsymbol{F}\boldsymbol{a}\boldsymbol{l}\boldsymbol{s}\boldsymbol{e}\:\boldsymbol{p}\boldsymbol{o}\boldsymbol{s}\boldsymbol{i}\boldsymbol{t}\boldsymbol{i}\boldsymbol{v}\boldsymbol{e}+\boldsymbol{T}\boldsymbol{r}\boldsymbol{u}\boldsymbol{e}\:\boldsymbol{n}\boldsymbol{e}\boldsymbol{g}\boldsymbol{a}\boldsymbol{t}\boldsymbol{i}\boldsymbol{v}\boldsymbol{e}+\boldsymbol{F}\boldsymbol{a}\boldsymbol{l}\boldsymbol{s}\boldsymbol{e}\:\boldsymbol{n}\boldsymbol{e}\boldsymbol{g}\boldsymbol{a}\boldsymbol{t}\boldsymbol{i}\boldsymbol{v}\boldsymbol{e}}\end{array}$$

## Pre-training with healthy and pathological kinematic datasets for VS classification

To evaluate the impact of transfer learning on our task, we pre-trained the model using two external datasets before fine-tuning it with the original dataset used in our baseline. These datasets were selected as they shared commonalities with our baseline, an important part of transfer learning^50^. The first dataset, KU-HAR^21^ (Supp Table 1), is a publicly available dataset comprising six-dimensional kinematic recordings from 90 healthy participants performing 18 different activities (e.g., walking, jumping). According to its authors, all participants provided informed consent permitting the use of anonymized data for research, with an option to withdraw participation. From the performed activities, we selected the “Walking for 20m” and “Running for 20m” tasks, as they resembled our walking tasks the most. In contrast to our dataset, KU-HAR only includes data from a single sensor which was positioned on the front of the waist. This dataset contains 20,750 samples, with each sample representing 3 s of non-overlapping activity, sampled at 100 Hz (yielding 300 time points per sample). Importantly, this data was not segmented into gait cycles. To match the format of our input data set, we similarly applied cubic interpolation to resample each sequence to 512 time points. Normalization was performed as described above for our dataset, using the value range of the training set for each iteration. We utilized only the regular walking and running activities from the KU-HAR dataset (882 and 595 samples, respectively), thereby creating a binary classification task with 1,477 total samples. This approach allowed us to assess the benefits of pre-training on a large, healthy, task-agnostic dataset prior to fine-tuning for VS classification.

The second dataset, the SCDS/Ataxia dataset (Supp Table 2), was drawn from an internal institutional database and collected under the same recording conditions as the VS dataset. It includes subjects diagnosed with cerebellar ataxia (28 subjects), superior canal dehiscence syndrome (SCDS; 16 subjects), and a small control group of healthy individuals (13 subjects). Aside from the differences in pathological diagnosis, all acquisition parameters—including sampling rate, dimensionality, and sensor placement—were identical to those used for the VS dataset. The same preprocessing steps (segmentation, normalization, resampling, etc.) were applied to prepare the data for model input. A binary classification task was defined between healthy controls and participants with vestibular-related conditions. For the walking on level ground task, the resulting dataset comprised 313 samples (57 from control and 256 from SCDS/Ataxia participants). This approach allowed us to assess the impact of pre-training on a smaller, task-specific dataset drawn from a population with relevant vestibular disorders.

Because the SCDS/Ataxia dataset only included recordings of the short-duration walking on level ground (task 1 of the FGA scale), and the KU-HAR walking/running sequences only spanned ~ 12 s per trial, these pre-training experiments were limited to the walking on level ground task. Sample distributions for each dataset are detailed in Table 3. Pre-training with the SCDS/Ataxia dataset used the same parameters as the baseline training, whereas pre-training with KU-HAR used a learning rate of 0.001. This lower learning rate allowed for smaller, and thus more precise, updates to the weights to accommodate for the larger difference in the pre-trained and original datasets. After pre-training, the model weights were saved and used as the initial values for the fine-tuned model. During fine-tuning, the convolutional layers were frozen, and only the final fully connected layers were updated over 20 epochs using the original dataset.

**Table 3:** Distribution of subjects and samples by pre-training external dataset.

Dataset	Type	#subjects	Task	#samples	% of samples
KU-HAR	Healthy	90	All	1477	100.00
	Healthy	90	Walking for 20 m	882	59.72
	Healthy	90	Running for 20 m	595	40.28
SCDS/Ataxia	All	57	Walking on level ground	313	100.00
	Control	13	Walking on level ground	57	18.21
	SCDS/Ataxia	44	Walking on level ground	256	81.79

## Results

To assess the utility of machine learning (ML) in detecting vestibular dysfunction, we systematically evaluated model performance under a range of clinically relevant conditions. We first established a baseline by testing classification accuracy using data from both gait tasks, walking on level ground (< 10 s) and walking with slow blinks (30s), revealing that the ML model can distinguish vestibular schwannoma (VS) patients from controls even in the absence of clinically detectable differences. We then assessed the contribution of individual sensor locations, the effect of pre-training on external datasets, and the impact of dataset composition in terms of subject and sample size. Together, these analyses identify critical parameters—sensor placement, task duration, training data characteristics—that optimize model performance and highlight the potential of ML to augment conventional clinical assessments.

## Machine learning reveals group differences undetected by clinical assessment

To obtain a baseline, we first evaluated model performance using samples from both the walking with eyes open (< 10 s) task and the extended task of walking with slow blinks, independently, using input from a single sensor for each run. This approach enabled us to assess the model’s ability to classify input signals based on sensor placement as well as examine how task duration influences classification accuracy. Classification accuracy averaged 0.623 and peaked at 0.737 for controls, and averaged 0.623 and peaked at 0.713 for VS participants (Table 4). Given that a value of 0.5 corresponds to random chance in this binary classification task, these results confirm meaningful discrimination. Moreover, the comparable performance across groups further indicates that the model is not biased toward either class. Confusion matrices, as well as F1 scores for all models going forward, can be found in the Supplemental Materials.

**Table 4:** Average accuracy from LOOCV by task, sensor, and subject group. In bold largest value for that task and that column.

Task	Sensor	Control	VS	Average
Walking on level ground (< 10 s)	Ankle (left)	0.6337	0.5907	0.6122
	Ankle (right)	0.5213	0.5903	0.5558
	Waist	0.5602	0.5119	0.5361
	Back	0.6122	0.6437	0.6280
	Head	0.5860	0.6215	0.6038
	Wrist	**0.6896**	**0.7132**	**0.7014**
	Mean	0.6005	0.6119	0.6062
Walking with slow blinks(30s)	Ankle (left)	0.6241	0.6138	0.6190
	Ankle (right)	0.6493	0.6062	0.6278
	Waist	0.5980	0.6469	0.6225
	Back	0.6577	0.6711	0.6644
	Head	0.6132	0.5834	0.5983
	Wrist	**0.7369**	**0.6868**	**0.7119**
	Mean	0.6465	0.6347	0.6406
Average of both tasks	Mean	0.6235	0.6233	0.6234

**Fig. 5:**
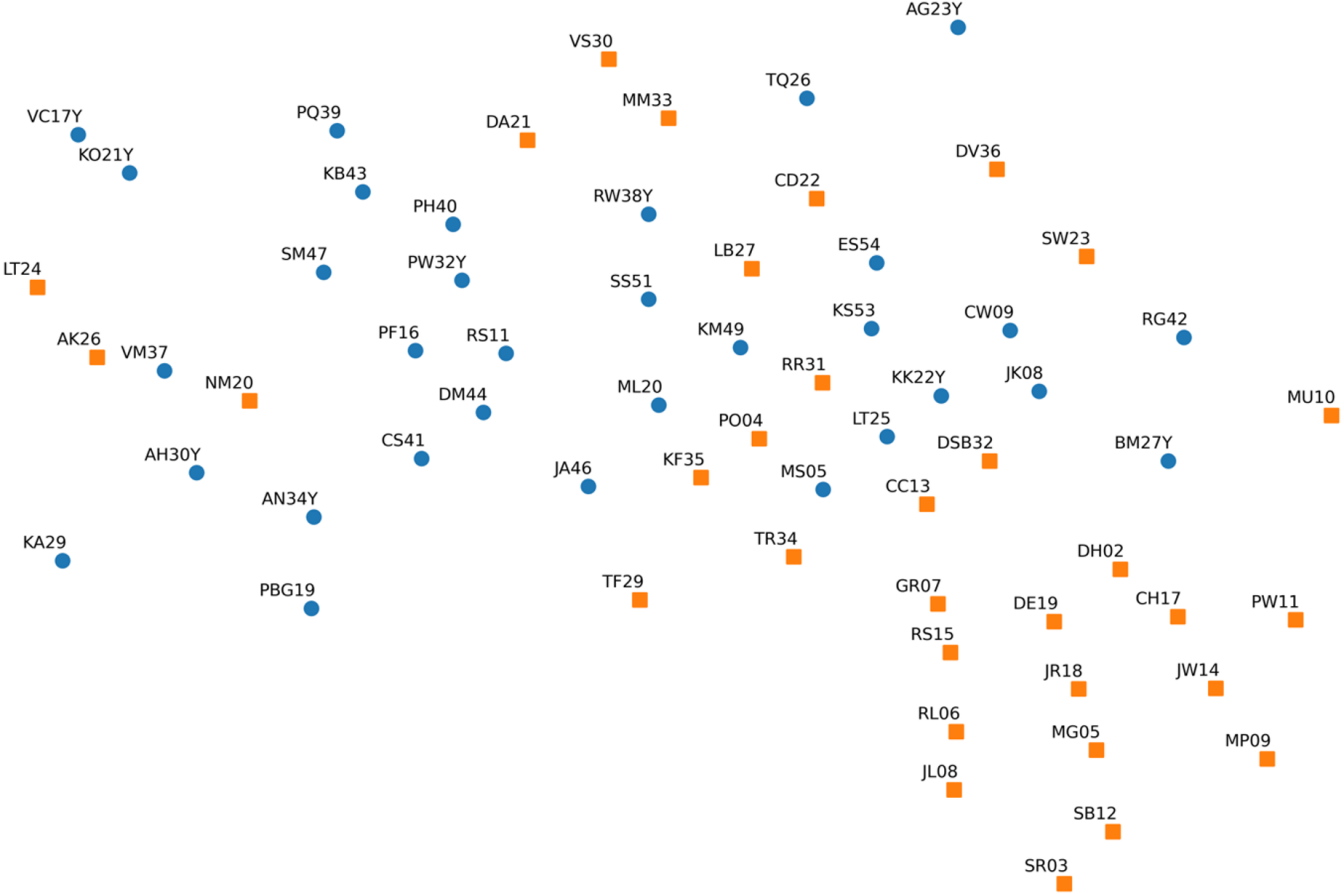
Representation of our dataset in two dimensions using t-SNE, with dynamic time warping as a distance metric. Blue circles: controls; orange squares: VS subjects. Each point represents the entire trial of a subject during the walking with eye open (< 10 s) task with data from the wrist sensor.

Importantly, we observed a modest increase in overall accuracy when using data from the extended gait task (accuracy improved from 0.606 to 0.641 when switching tasks; Table 4), highlighting the benefit of training on a larger dataset (312 samples for walking on level ground (< 10 s) versus 1,393 for the extended gait task of walking with slow blinks (30s); Table 2). Visualization of the dataset using t-SNE, a tool for dimensionality reduction and low-dimensional visualization (Fig. 5), revealed that although the data are not linearly separable, control and VS samples form distinct clusters—explaining the model’s capacity to differentiate between groups despite overlap in clinical assessments.

In clinical practice, FGA tasks are evaluated by trained experts on a standardized scale ranging from 0 to 3, with higher scores indicating better performance and faster completion. For the walking on level ground task, clinical scores were available for both control and VS participants, allowing us to directly compare the distribution of ratings across groups (Table 5). The resulting distributions were highly similar: control participants received an average score of 2.81 ± 0.40, while VS participants scored 2.81 ± 0.47. This close alignment in clinical ratings suggests that, from a clinician’s perspective, the two groups perform equivalently on this task. However, despite the lack of observable group-level differences in clinical assessment, our machine learning model was still able to achieve classification of VS and control participants with a peak average accuracy of 0.701 (Table 4). This finding highlights the added value of the ML approach in revealing subtle distinctions that may elude standard clinical evaluation.

**Table 5:** Distribution of FGA1 clinical score per group for walking on level ground (*<* 10 s). Possible values for scores are integers between 0 and 3, 3 being the best possible score, both included. For each score, the number of subjects of a given group, and the percentage that represents within their group, is shown. Average score ± standard deviation included per group.

Score	Control	VS
0	0 (0%)	0 (0%)
1	0 (0%)	1 (3%)
2	6 (19%)	4 (13%)
3	26 (81%)	27 (84%)
Average	2.81 *±* 0.40	2.81 *±* 0.047

## Sensor placement strongly influences model performance: wrist sensor leads

When evaluating the classification performance of each individual sensor across both tasks, the wrist sensor consistently produced the highest accuracy—outperforming all other sensor placements by a substantial margin. For the walking on level ground task, the wrist sensor achieved an average accuracy of 0.701 across the full dataset, while the mean accuracy across all sensors was 0.6062, making the wrist sensor’s performance 0.095 above average. In comparison, the second-best performing sensor—the back sensor—reached an average accuracy of 0.6280, only 0.0218 above the average. A similar pattern was observed for the extended gait exercise. The wrist sensor achieved an average accuracy of 0.7119, compared to a mean accuracy of 0.641 across all sensors—an improvement of 0.071. Again, the back sensor ranked second, with an average accuracy of 0.664, just 0.024 above the mean. These results, detailed in Table 4, underscore the consistent advantage of the wrist sensor across both tasks.

**Fig. 6:**
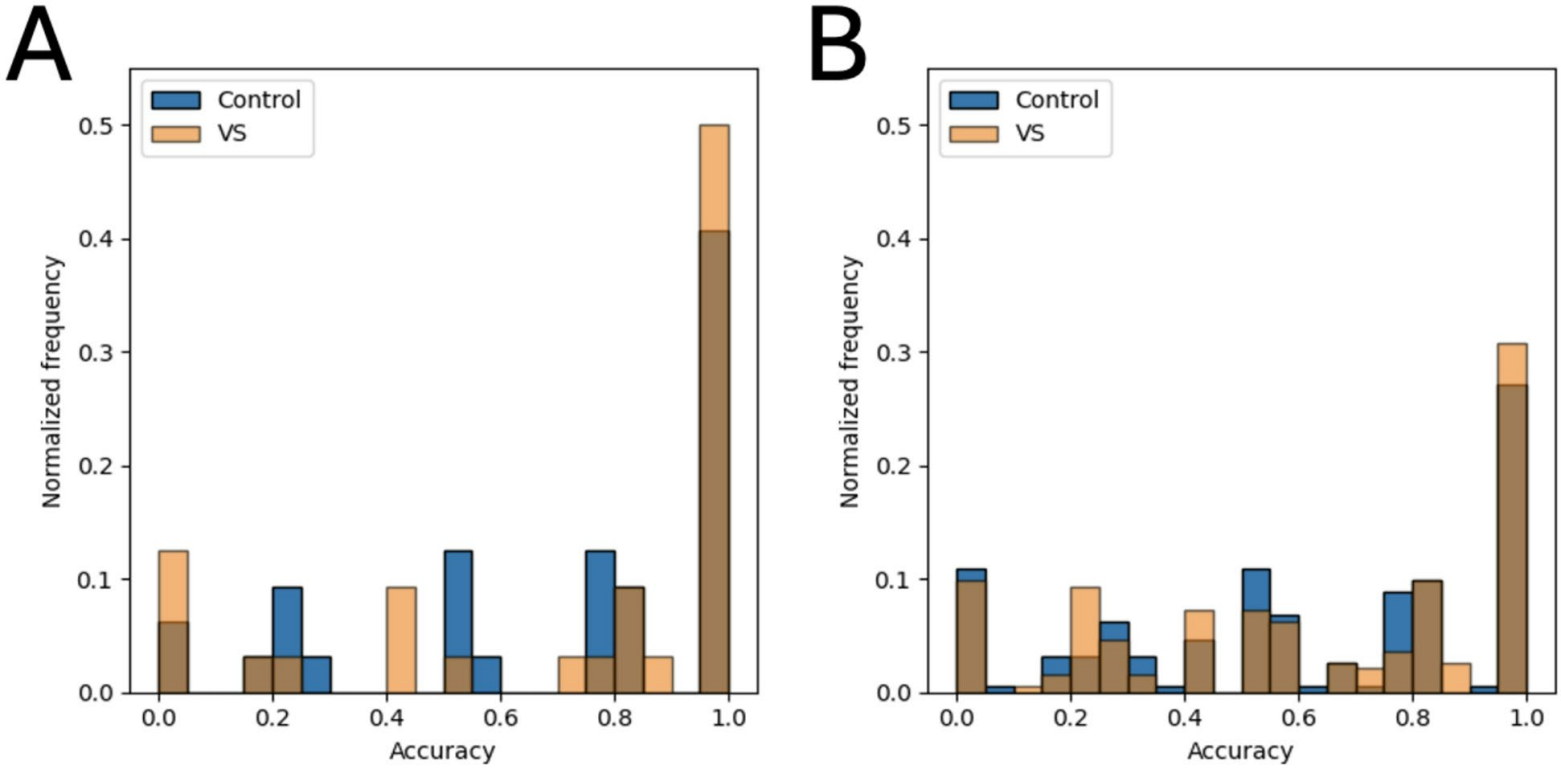
Histograms of accuracy per subject on a normalized frequency. (**A**) Values for the performance with wrist sensor. (**B**) Values for the average performance of all sensors. Both (A) and (B) correspond to accuracy results from the walking on level ground (*<* 10 s) task.

To further illustrate the impact of sensor choice, we visualized classification accuracy per subject using histograms normalized by frequency for the walking on level ground task (Fig. 6). This comparison showed that the wrist sensor achieved perfect classification, i.e. it classified all of the participants cycles correctly, for over 40% of both control and VS participants, whereas this figure hovered around 30% when averaging across all sensors. These results not only confirm that the wrist sensor delivers superior performance but also demonstrate its unique ability to perfectly classify a larger proportion of individual subjects.

### Pre-training on disease-specific vs. healthy-only datasets yields distinct outcomes

To assess the impact of transfer learning on classification performance, we implemented two pre-training strategies prior to fine-tuning on the VS dataset. Specifically, we used the external KU-HAR dataset and our internal SCDS/Ataxia dataset (see Methods). This pre-training was conducted using data from the walking on level ground task only, as this task more closely resembled the structure of the pre-training datasets. Notably, the SCDS/Ataxia dataset does not contain data from the extended gait task, further motivating this focus.

Model performance under each pre-training condition was evaluated using multiple folds of validation data. For both SCDS/Ataxia and VS datasets, we computed the mean of average subject-level accuracies across validation folds. In contrast, because KU-HAR is not divided by subject but rather by task, its metric was the mean accuracy across all validation samples. Importantly, the data included in each validation fold was entirely independent from the data used in the other folds, ensuring no overlap during training and evaluation. As summarized in Table 6, both models successfully learned meaningful features from their respective datasets, evidenced by validation accuracies of 0.718 for SCDS/Ataxia and 0.88 for KU-HAR. The lower performance observed for SCDS/Ataxia likely reflects greater subject-level variability (including healthy, SCDS, and ataxia participants) compared to KU-HAR (exclusively healthy individuals). Nonetheless, surpassing 0.7 accuracy demonstrates the promise of such datasets for training models to detect a broader range of vestibular dysfunction, including but not limited to VS, and suggests directions for future work.

**Table 6:** Results from KU-HAR and SCDS/Ataxia datasets on the pre-training task, performed before fine-tuning the model with the VS data.

Dataset	#folds	Sensor	Performance
KU-HAR	10	n/a	0.8796
SCDS/Ataxia	7	Ankle (left)	0.7163
		Ankle (right)	0.6744
		Waist	0.6986
		Back	0.6967
		Head	0.7600
		Wrist	0.7618
		Mean	0.7180

Having confirmed that our model could effectively extract relevant features from both pre-training datasets, next we fine-tuned the model on the VS dataset and compared performance to the original baseline. Results (Table 7) showed that the effect of pre-training was highly dependent on the dataset used. Pre-training with the KU-HAR dataset led to modest improvements in accuracy for control participants (average increase of 0.038) but produced a performance decline for VS subjects (average decrease of 0.088). In striking contrast, pre-training with the SCDS/Ataxia dataset yielded performance gains for both groups: an average increase of 0.1 for controls and 0.064 for VS subjects.

**Table 7:** Results from pre-training scenarios and difference with corresponding baseline results (added in parenthesis) for walking on level ground, by sensor, and group.

Pre-training Scenario	Sensor	Control	VS
KU-HAR dataset	Ankle (left)	0.6107 (−0.0230)	0.5684 (−0.0223)
	Ankle (right)	0.5643 (+ 0.0430)	0.4172 (−0.1731)
	Waist	0.6395 (+ 0.0793)	0.5344 (+ 0.0225)
	Back	0.7005 (+ 0.0883)	0.5737 (−0.0700)
	Head	0.5506 (−0.0354)	0.4472 (−0.1743)
	Wrist	0.7639 (+ 0.0743)	0.6024 (−0.1108)
	Mean	0.6383 (+ 0.0378)	0.5239 (−0.0880)
SCDS/Ataxia dataset	Ankle (left)	0.7604 (+ 0.1267)	0.7118 (+ 0.1211)
	Ankle (right)	0.6427 (+ 0.1214)	0.6422 (+ 0.0519)
	Waist	0.7080 (+ 0.1478)	0.6376 (+ 0.1257)
	Back	0.6762 (+ 0.0640)	0.6778 (+ 0.0341)
	Head	0.6498 (+ 0.0638)	0.6198 (−0.0017)
	Wrist	0.7646 (+ 0.0750)	0.7684 (+ 0.0552)
	Mean	0.7003 (+ 0.0998)	0.6763 (+ 0.0644)

Sensor-specific effects were also observed. For the KU-HAR pre-training condition, the largest improvements were noted for the waist and back sensors—those most closely aligned with KU-HAR’s sensor placement at the front of the waist—suggesting sensor alignment plays a critical role in transfer effectiveness. Importantly, the wrist sensor continued to outperform all others in both pre-training conditions. However, in the SCDS/Ataxia case, the performance gap between the wrist sensor and the next-best sensor (ankle left) was reduced, as all sensor placements benefited from pre-training. Together, these findings indicate that KU-HAR pre-training yields localized, sensor-specific gains driven by spatial alignment, whereas SCDS/Ataxia pre-training provides broader, generalized improvements arising from shared pathological gait features and consistent recording conditions. These results support the conclusion that the effectiveness of transfer learning depends primarily on the physiological relevance of the source dataset rather than its size or task similarity.

### Recruiting more subjects improves classification more than extending individual recordings

To extend the clinical utility of our findings, we next investigated which dataset-level variables have the greatest influence on model performance. Specifically, we examined how the number of subjects versus the number of samples per subject impact classification accuracy—insights that can inform the design of future experimental protocols, in the same manner as our above analysis of sensor placement.

We first evaluated how model accuracy varies with different numbers of subjects per class (25%, 50%, and 75%), using data from the wrist sensor, which had previously shown the highest classification accuracy (Table 4). This analysis was performed for both the walking on level ground and extended gait tasks. It builds upon our baseline model, which included 32 subjects per group (31 for the extended gait task). To account for randomness in subject selection, we performed 15 iterations for each condition and reported both the mean classification accuracy and standard deviation across folds (Table 8). It is important to note that different subjects contributed varying numbers of cycles per task, and all cycles were retained to preserve within-subject variability—we did not normalize the number of samples per subject. Reducing the number of subjects led to a pronounced decline in model performance. For the walking on level ground task, accuracy dropped from a baseline of 0.701 to 0.617 (a decrease of 0.084) when only 25% of subjects were used. Similarly, for the extended gait task, performance dropped from 0.712 to 0.65 (a decrease of 0.062). These reductions were accompanied by increased variability in model performance across iterations, with standard deviations peaking at 0.141 for walking on level ground and 0.118 for extended gait, reflecting the instability introduced by smaller subject pools.

**Table 8:** Dependency on the #subjects on wrist sensor by task among the entire dataset (#iterations = 15, mean *±* standard deviation).

Task	% subjects per class (*n*)	Average performance
Walking on level ground (*<* 10 s)	25 (8)	0.6169 *±* 0.1415
	50 (16)	0.6846 *±* 0.0764
	75 (24)	0.6859 *±* 0.0460
	Baseline (32)	0.7014
Walking with slow blinks (30s)	25 (8)	0.6498 *±* 0.1177
	50 (16)	0.6626 *±* 0.0599
	75 (24)	0.6893 *±* 0.0468
	Baseline (31)	0.7119

We next asked whether reducing the number of samples per subject would yield a similar effect. Using the full subject pool, we retained only 25%, 50%, or 75% of each participant’s samples (i.e., cycles), thereby reducing intra-subject variability while maintaining inter-subject diversity. Again, using the wrist sensor for both tasks, we conducted 15 iterations of LOOCV per condition and reported mean accuracy and standard deviation (Table 9). In contrast to the subject-count manipulation, restricting the number of samples per subject produced only modest performance declines. For the walking on level ground task, accuracy fell from 0.701 to 0.658 (a decrease of 0.043); for the extended gait task, accuracy declined slightly from 0.712 to 0.7 (a change of just 0.012). Variability across iterations also remained more stable—particularly for the extended gait task, where the standard deviation never exceeded 0.048.

**Table 9:** Dependency on the #samples on wrist sensor by task among the entire dataset (#iterations = 15, mean *±* standard deviation).

Task	% samples per subject	Average performance
Walking on level ground (*<* 10 s)	25	0.6580 *±* 0.0945
	50	0.6723 *±* 0.0540
	75	0.6816 *±* 0.0367
	Baseline (100)	0.7014
Walking with slow blinks (30s)	25	0.7002 *±* 0.0483
	50	0.7064 *±* 0.0315
	75	0.7041 *±* 0.0262
	Baseline (100)	0.7119

**Fig. 7:**
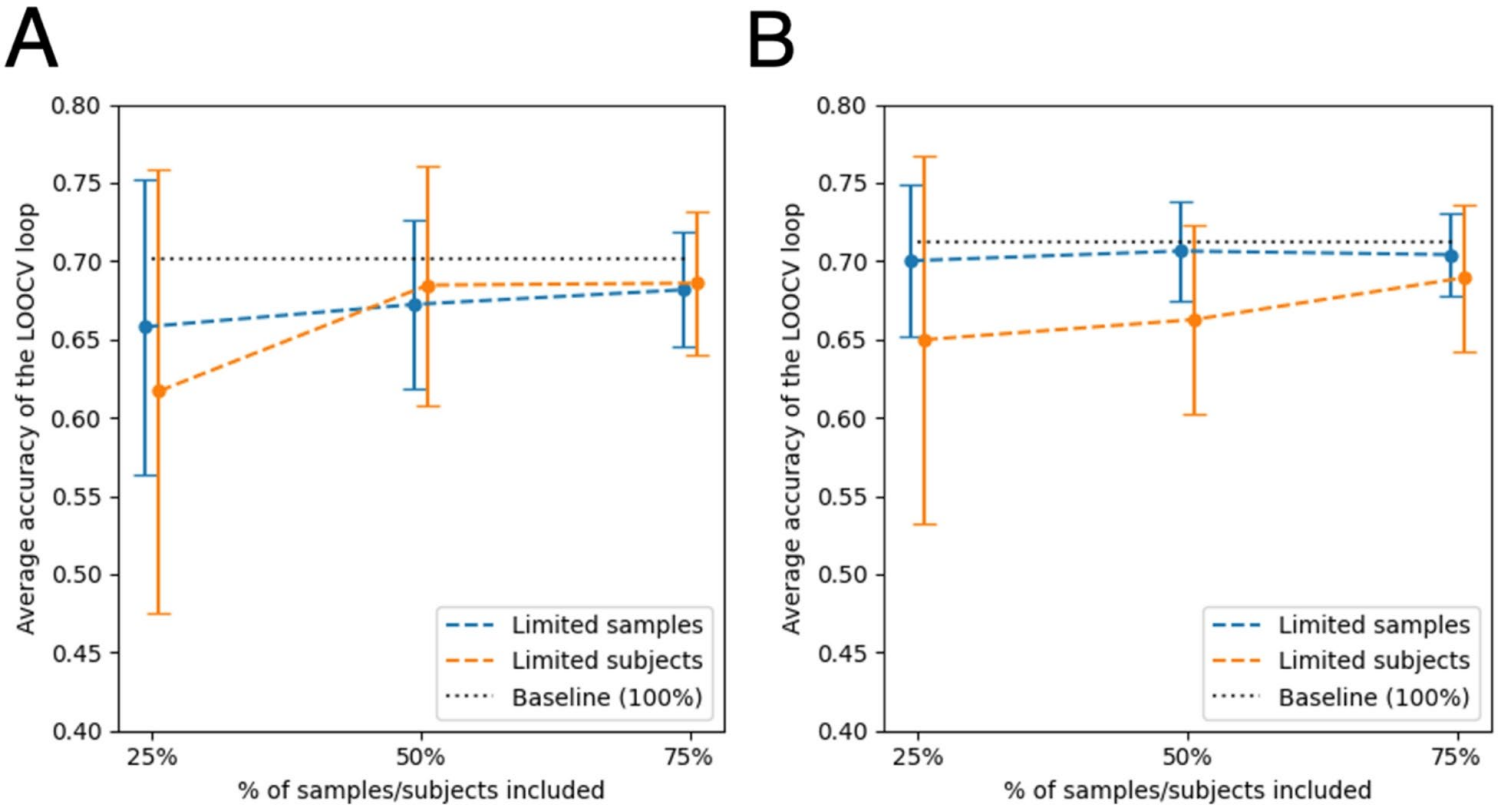
Change in performance when limiting the percentage of samples or subjects compared to the baseline. (**A**) Values for the walking on level ground (*<* 10 s) task. (**B**) Values for the extended, walking with slow blinks, gait exercise (30s). Both (A) and (B) include mean *±* standard deviation.

Finally, to directly compare the effects of subject versus sample count, we plotted the results from Tables 8 and 9 alongside baseline performance metrics (Fig. 7). For the walking on level ground task, both subject and sample reductions negatively affected model accuracy. In contrast, for the extended gait task, only reductions in subject count impacted performance—limiting the number of samples had little effect. This pattern suggests that the extended task may exceed a critical threshold for within-subject data volume, beyond which additional samples offer diminishing returns. However, our sample of 64 total subjects appears insufficient to reach a similar threshold for subject diversity. These findings underscore that, when aiming to improve model robustness for clinical use, expanding the number of participants is more beneficial than increasing the volume of data collected per individual.

Together, these results demonstrate that model performance is most strongly influenced by subject diversity, sensor placement, and task duration—factors that should be carefully considered when designing future studies aimed at leveraging machine learning for clinical gait analysis and vestibular dysfunction detection.

## Discussion

In this study, we demonstrate that deep learning models trained on wearable sensor data can effectively distinguish vestibular schwannoma (VS) patients from healthy controls, even when standard clinical scoring failed to reveal differences. All data were collected prior to surgical intervention, providing a rare opportunity to detect subclinical motor adaptations before permanent vestibular loss. Importantly, our model identified impairments during minimally challenging gait tasks, highlighting its sensitivity to early-stage compensatory strategies. Our findings reveal three key insights: (1) body mounted IMU data—especially from the wrist—contain discriminative kinematic signatures of vestibular dysfunction; (2) classification accuracy improves with greater subject diversity, supporting the importance of expanding cohort size over increasing per-subject data; and (3) transfer learning with disease-specific datasets meaningfully enhances model performance, underscoring the value of leveraging relevant vestibular pathologies to improve generalization. Together, these findings highlight the promise of deep learning and wearable sensors as scalable, objective tools for detecting subtle dysfunction and supporting earlier diagnosis and monitoring in VS and related disorders.

### Model performance across tasks and sensors

We developed a machine learning model capable of successfully classifying kinematic data from individuals with vestibular schwannoma (VS) and age-matched healthy controls. Classification performance was evaluated across two conditions: a standard ~ 10-second FGA walking task (walking on level ground, eyes open) and an extended 30-second version (walking with eyes closed every 2 steps). Both tasks yielded comparable results, with slightly higher accuracy observed during the extended task. This finding is notable given our previous report that head kinematics vary with task duration, showing greater range of motion during more challenging, prolonged walking exercises^51^. Nevertheless, the model effectively extracted relevant features from both conditions, suggesting robustness to natural variability in movement dynamics.

Despite the modest size of our dataset - a common constraint in clinical machine learning studies - our model achieved strong classification performance, reaching peak accuracies of 0.712 (extended gait, 30 s) and 0.701 (walking on level ground, 10 s) when using data from a single wrist-mounted sensor. This is notable in the context of prior work demonstrating kinematic differences between VS patients and controls^5,6,41,51,52^—differences our model was able to detect and leverage. Among all tested sensor placements, the wrist consistently outperformed others, indicating its potential as an optimal site for wearable monitoring. While future validation with additional tasks is warranted, these findings underscore the feasibility of high-accuracy classification using a lightweight, user-friendly sensor setup. Importantly, in contrast to the manual clinical scores, which were uniformly high and failed to differentiate between groups, our model achieved over 70% accuracy in classifying VS patients and controls. This suggests that even simple, wrist-based kinematic data can capture subtle impairments that elude conventional clinical scoring—underscoring the added value of data-driven approaches for detecting vestibular dysfunction.

Although individual wrist motion parameters are difficult to extract from the low-interpretability CNN model, our findings demonstrate the model’s potential and support further investigation of wrist-derived signals as a valuable source of information on movement patterns in individuals with VS. Future work could include post-hoc analyses of raw wrist-sensor signals (e.g., Supplementary Fig. 2) to identify where the key differences arise. The head sensor’s weaker performance may reflect the fact that, although patients often restrict head motion^51^ a feature that differentiates them from controls—reduced motion itself provides limited discriminatory information. Our decision to train separate models for each sensor was intended to isolate and evaluate the individual contribution of each signal; however, combining all sensors in an ensemble framework could further enhance classification accuracy. We note this as an important direction for future research while acknowledging the clinical trade-off between maximizing model performance and maintaining ease of implementation. Moreover, the present study focused on only two gait tasks. While the wrist sensor yielded superior performance in this context, this advantage may partly reflect task selection. Future work should therefore vary task type, speed, and distance metrics to determine how these factors influence each sensor’s contribution. Incorporating more challenging conditions from the Functional Gait Assessment—such as walking with head turns, on a narrow base, or with eyes closed—could provide a more comprehensive evaluation of sensor-specific performance and generalization across behaviors.

### Transfer learning and the value of Pathology-Relevant data

Overall, we found that pre-training our model on a clinically relevant dataset—specifically, kinematic recordings from individuals with cerebellar ataxia and superior canal dehiscence syndrome (SCDS)—substantially improved classification accuracy for both VS patients and healthy controls, with performance exceeding 0.76 (Table 7). In contrast, pre-training on the much larger KU-HAR dataset, which included only healthy individuals performing general activities, had negligible or even negative effects on accuracy. These results highlight the importance of pathology-relevant data in clinical machine learning. While the SCDS/Ataxia dataset encompassed diverse vestibular-related and other neurological impairments, it appears that shared features of the kinematic data exist across patient groups that enabled the model to learn transferable representations of abnormal gait—something not achievable with the KU-HAR dataset, which included only data from healthy individuals. This contrast suggests that, in our case, the clinical relevance of the pre-training dataset had a greater impact on model performance than its size. It is also important to note that preprocessing steps differed between the two datasets, particularly in the segmentation stage. While the SCDS/Ataxia dataset followed the same pipeline as our original data, with recordings segmented into gait cycles, the KU-HAR dataset did not include segmentation. These methodological differences likely contributed to the observed performance discrepancies. While further validation is needed, our findings suggest that smaller, well-curated datasets reflecting the target pathology - and processed using comparable preprocessing pipelines- may yield greater benefits for model generalization than larger, non-specific datasets.

### Data collection considerations for clinical ML applications

To inform future study design, we examined how reducing either the number of subjects or the number of samples per subject influenced model performance. Reducing the subject pool led to significant declines in accuracy and increased variability, emphasizing the importance of participant diversity. In contrast, limiting the number of gait cycles per subject had only modest effects, particularly during the extended 30-second task. This suggests that longer tasks provide sufficient data to characterize individual movement patterns, allowing reliable classification with fewer repeated trials. Our findings point to a practical tradeoff: increasing the number of participants yields greater benefits than collecting more data per individual. Encouragingly, a 30-second walking task appears to capture meaningful within-subject variability without placing excessive burden on patients or clinicians.

Our findings highlight a key advantage of using deep learning—specifically, a convolutional neural network (CNN)—to analyze raw kinematic data. Unlike traditional methods that rely on hand-crafted features (for example, gait cycle mean length or amplitude^5,51,52^. CNNs learn relevant patterns directly from the data without requiring domain-specific feature engineering. This offers an important advantage in clinical contexts, where symptom presentation may vary across individuals and relevant features may not be obvious or consistently defined. This type of model has been applied to gait classification tasks and has shown considerable promise due to its ability to leverage automated feature extraction and hierarchical representation learning^42–45^. Our lower accuracies compared to these models likely reflect the greater difficulty of our task, as our patient cohort shares a single underlying pathology.

This deep learning approach contrasts with prior work by Jabri et al.^22^, where 44 spatiotemporal features—including angular velocity, acceleration, stride length, and stride time—were manually extracted and used to train a random forest classifier. Moreover, although previous studies have reported success using alternative model architectures, such as random forests (^23^Hope et al.), we deliberately avoided manual feature selection strategy to minimize potential bias, which can arise when chosen features fail to capture key aspects of pathology or differ in relevance across patient subgroups. Notably, our classification task was especially challenging given the limited sample size and heterogeneity of VS symptoms. However, by focusing on a single diagnostic category, our model better reflects real-world clinical settings, where algorithms must generalize across individuals with varying impairments. In future work, unsupervised learning could help identify clinically meaningful subgroups within the VS population. For example, Tarnutzer et al.^53^ demonstrated that stratifying patients based on vestibulo-ocular reflex (VOR) measures can support more personalized rehabilitation strategies^29^.

### Clinical utility and the case for quantitative gait biomarkers

As previously noted, our approach is not intended to replace current diagnostic methods. However, it holds potential for guiding personalized rehabilitation and informing clinical decisions, particularly early in disease progression. Because our cohort included patients with vestibular schwannoma (VS) prior to tumor removal surgery, our findings highlight the potential to assess functional status and monitor progression during the preoperative period. Clinicians may find this result counterintuitive, given the standard reliance on subjective rather than objective measures for movement characterization. However, we were similarly surprised in our previous work, where patients with VS scored within the normal range on clinical balance assessments yet exhibited clear gait kinematic abnormalities when objectively evaluated^5,6,41,51,52^.

Building on this prior work, we found that our current method was more sensitive to differences between groups than standard measures such as subjective FGA scoring^7^—even in the relatively undemanding walking on level ground, FGA 1 task. We speculate that models trained on more challenging tasks may perform even better. This is supported by the fact that the extended task included intermittent eye closure, which can be slightly more challenging than normal level-ground walking with eyes open, even if their durations were the same. Thus, introducing a mild and tolerable challenge to normal walking, along with longer task duration, may both contribute to improved task performance. Standard FGA scoring is limited by both observer-based assessment and a narrow integer scale. In contrast, our model provides a continuous output from zero to one. While we applied a 0.5 threshold for classification in this study, future work could leverage the raw output as a finer-grained index of model certainty. Furthermore, kinematic data captures movement features beyond what can be discerned by human observation, as evidenced by the model’s high accuracy. We anticipate that IMU-based testing will help refine testing choices regarding both task duration and task selection. Importantly, a model with a known accuracy can provide an objective performance metric to help clinicians evaluate its potential utility. This added transparency may improve the clinical interpretability of gait assessments and support more informed selection of predictive tools.

To support VS patient monitoring and inform decisions concerning surgical intervention, there is a need for tools that can track changes in vestibular function over time. Our findings suggest that combining kinematic data with traditional clinical scores may help address this gap. In prior work, we observed that subjective measures correlated poorly with both clinical status^5^ and tumor size^54^, underscoring the limitations of current assessment approaches. Earlier versions of our kinematic score^5,6,51,54^ distinguished among healthy controls, preoperative, and postoperative VS patients. The present model builds on that framework, offering improved classification of VS patients versus healthy individuals. Given the variability in symptom presentation across patients, this approach may also be useful for longitudinal tracking within individuals.

Indeed, over time or with rehabilitation, a patient’s status may shift towards a healthier profile – changes that our model could potentially detect. Moreover, the continuous output of the classifier could be explored in future studies as the basis for a novel kinematic scoring system, offering a more detailed characterization of patient movements. In addition, the model’s known accuracy provides a quantifiable benchmark that could help clinicians assess its utility in a given setting. By increasing the objectivity and sensitivity of gait assessment, this tool may enhance the clinical relevance of test batteries used to evaluate vestibular dysfunction, a goal that has been pursued in other disease domains^55–57^. Although the current model has not been validated for distinguishing between different levels of disease severity, our findings are promising and warrent further investigation in this direction. Improving model accuracy remains equally important, as the current performance levels may still be insufficient for clinical deployment.

### Personalized prehabilitation and future research directions

Prior to surgery, patients with vestibular schwannoma often develop abnormal patterns of gait and head motion, which we have previously characterized in detail^6,51,54^. Moreover, following vestibular nerve resection, patients tend to revert to these maladaptive motor states^6,51,54^. This suggests that preventive strategies aimed at minimizing the development or reinforcement of such abnormal behaviors could improve long-term outcomes. While current standards of care typically focus on rehabilitation after surgery, there is growing evidence that prehabilitation (“prehab”)—targeted interventions implemented before surgery—can reduce symptoms and improve recovery^58,59^. For example, recent prehab approaches for vestibular patients have included balance and gaze-stabilization training prior to surgery, with the goal of reducing postoperative dizziness and unsteady.

Our modeling approach offers a potential biomarker for monitoring disease progression and evaluating therapy response in VS patients by quantifying gait deviations relative to healthy controls. While the model functions as a ‘black box’, prior analyses of the underlying kinematic data have revealed consistent group-level differences, lending interpretability to its outputs. To further increase interpretability, future work could incorporate explainable artificial intelligence (AI) approaches^60^ to identify specific kinematic features that can distinguish between groups. Future studies should explore the integration of complementary clinical measures to help explain inter-subject variability and assess the model’s added value. Expanding the analysis to additional FGA tasks and longitudinally tracking patients during rehabilitation or post-surgical recovery will be important next steps for evaluating its clinical utility. In parallel, future work should examine how variations in tumor size and location -as well as proximity to peripheral vestibular organs, associated impairments, and sensory reweighting differences- affect ML model performance. Ideally, subsequent models should account for these factors to advance a more personalized medicine approach.

Finally, architectures such as recurrent neural networks (RNNs) and bilateral long short-term memory (Bi-LSTM) networks also show promise for handling sequential data and warrant evaluation in this context.

## Conclusion

Here, we have demonstrated that machine learning models trained on kinematic data recorded via wearable sensors can accurately classify VS patients, even when traditional clinical scores show no group differences. These models offer objective, high-performing tools that could augment current diagnostic workflows. Wrist-mounted sensors yielded the highest performance, supporting their use in both clinical and at-home settings. We also show that longer tasks (e.g., 30 s) reduce the need for repeated trials, and that increasing participant numbers has a greater impact on accuracy than increasing per-subject data. Finally, transfer learning with clinically relevant datasets enhanced model performance, underscoring the value of pathology-specific data. Together, these findings—derived from pre-surgical VS patients—lay the groundwork for future tools to support diagnosis, monitor progression, and guide personalized rehabilitation in vestibular disorders.

## Supplementary Information

Below is the link to the electronic supplementary material.

Supplementary Material 1

Supplementary Material 2

## Data Availability

Data may be made available on request to the corresponding author.
