# Supplementary material for: A deep learning pipeline for detecting vestibular schwannoma patients with unilateral vestibular loss based on kinematic data
Source: Sci Rep. 2025 Nov 25;15:45343. doi: 10.1038/s41598-025-29776-8 (PMC12748630; doi:10.1038/s41598-025-29776-8)
Supplement: Supplementary file 1 — Supplementary Material 1 [file 41598_2025_29776_MOESM1_ESM.docx]

Supplemental materials for A deep learning pipeline for detecting vestibular schwannoma patients with unilateral vestibular loss based on kinematic data

Supp Table 1: Dataset gender and age information for KU-HAR dataset.

| **Gender** | **Parameter** | **Subjects** |
| --- | --- | --- |
| All | Count | 90 |
|  | Mean Age | 21.7 |
|  | Age Range | 18-34 |
| Male | Count | 75 |
| Female | Count | 15 |

Supp Table 2: SCDS/Ataxia dataset gender and age information.

| **Gender** | **Parameter** | **Control** | **SCDS/Ataxia** |
| --- | --- | --- | --- |
| All | Count | 13 | 44 |
|  | Mean Age± SD (years) | 44.11*±*8.2 | 53.36*±*13.83 |
|  | Age Range | 33-56 | 22-84 |
| Male | Count | 6 | 17 |
|  | Mean Age± SD (years) | 42.33*±*8.33 | 52*±*17.71 |
|  | Age Range | 33-54 | 22-84 |
| Female | Count | 7 | 27 |
|  | Mean Age± SD (years) | 45.64*±*8.41 | 54.22*±*11 |
|  | Age Range | 33-56 | 38-72 |

SD: standard deviation.
