# Supplementary material for: A deep learning pipeline for detecting vestibular schwannoma patients with unilateral vestibular loss based on kinematic data
Source: Sci Rep. 2025 Nov 25;15:45343. doi: 10.1038/s41598-025-29776-8 (PMC12748630; doi:10.1038/s41598-025-29776-8)
Supplement: Supplementary file 2 — Supplementary Material 2 [file 41598_2025_29776_MOESM2_ESM.docx]

Supplemental materials for A deep learning pipeline for detecting vestibular schwannoma patients with unilateral vestibular loss based on kinematic data

Supp Table 1: Dataset gender and age information for KU-HAR dataset.

| **Gender** | **Parameter** | **Subjects** |
| --- | --- | --- |
| All | Count | 90 |
|  | Mean Age | 21.7 |
|  | Age Range | 18-34 |
| Male | Count | 75 |
| Female | Count | 15 |

Supp Table 2: SCDS/Ataxia dataset gender and age information.

| **Gender** | **Parameter** | **Control** | **SCDS/Ataxia** |
| --- | --- | --- | --- |
| All | Count | 13 | 44 |
|  | Mean Age± SD (years) | 44.11*±*8.2 | 53.36*±*13.83 |
|  | Age Range | 33-56 | 22-84 |
| Male | Count | 6 | 17 |
|  | Mean Age± SD (years) | 42.33*±*8.33 | 52*±*17.71 |
|  | Age Range | 33-54 | 22-84 |
| Female | Count | 7 | 27 |
|  | Mean Age± SD (years) | 45.64*±*8.41 | 54.22*±*11 |
|  | Age Range | 33-56 | 38-72 |

SD: standard deviation.


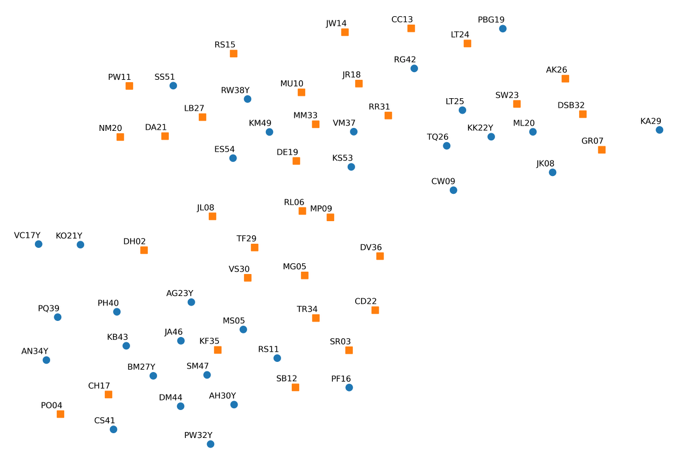
Supp Figure 1: Representation of our dataset in two dimensions using t-SNE, with dynamic time warping as a distance metric and trustworthiness (from the scikit-learn Python package) in parentheses measuring embedding quality. Blue points: controls; orange points: VS subjects. Each point represents the entire trial of a subject during the walking with eye open (<10s) task with data from all sensors: A) Ankle left (0.85), B) Ankle right (0.8), C) Wrist (0.65), D) Head (0.64), E) Back (0.61), F) Waist (0.63).

B

A


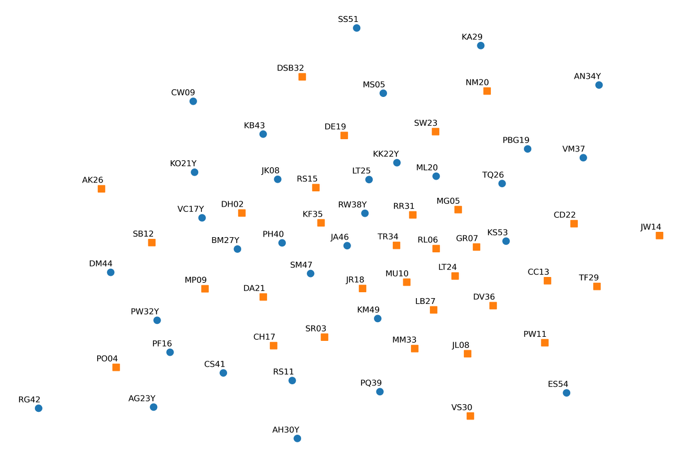

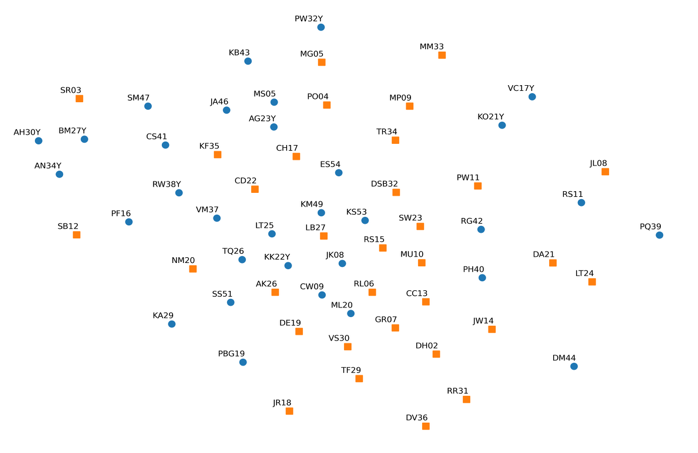

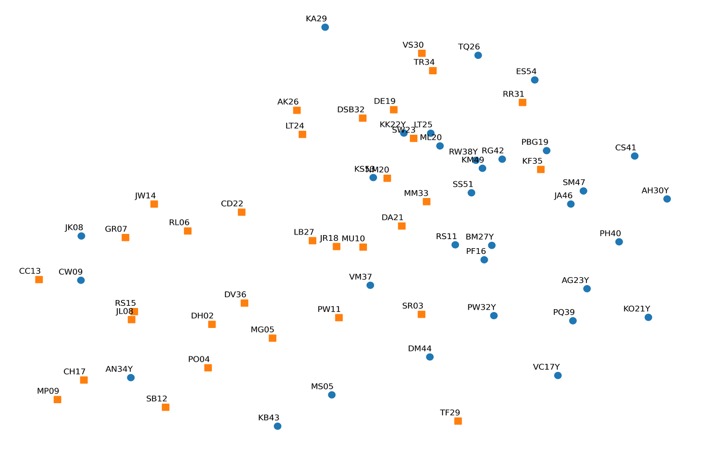


D

C

F

E


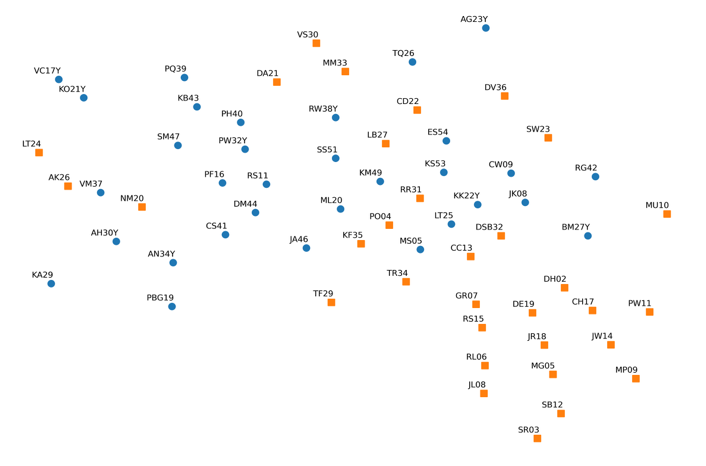

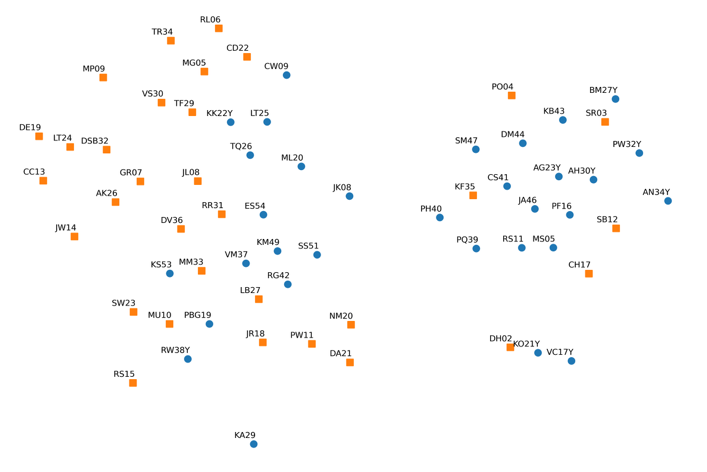


Supp Table 3: Confusion matrices and F1 scores for tasks from Table 4. Highest F1 scores are in bold.

| Task | Sensor | True positive | True negative | False positive | False negative | F1 score |
| --- | --- | --- | --- | --- | --- | --- |
| Walking on level ground (<10s) | Ankle (left) | 96 | 95 | 58 | 63 | 0.6134 |
|  | Ankle (right) | 96 | 79 | 74 | 63 | 0.5836 |
|  | Waist | 83 | 85 | 68 | 76 | 0.5355 |
|  | Back | 103 | 86 | 67 | 56 | 0.6261 |
|  | Head | 100 | 87 | 66 | 59 | 0.6154 |
|  | Wrist | 114 | 104 | 49 | 45 | **0.7081** |
|  | Mean |  |  |  |  | 0.6137 |
| Walking with slow blinks(30s) | Ankle (left) | 439 | 437 | 253 | 264 | 0.6294 |
|  | Ankle (right) | 419 | 450 | 240 | 284 | 0.6153 |
|  | Waist | 456 | 422 | 268 | 247 | 0.6391 |
|  | Back | 478 | 465 | 225 | 225 | 0.6800 |
|  | Head | 402 | 433 | 257 | 301 | 0.5903 |
|  | Wrist | 482 | 513 | 177 | 221 | **0.7078** |
|  | Mean |  |  |  |  | 0.6436 |

Supp Table 4: Confusion matrices and F1 scores for tasks from Table 7. Highest F1 scores are in bold.

| Pre-training Scenario | Sensor | True positive | True negative | False positive | False negative | F1 score |
| --- | --- | --- | --- | --- | --- | --- |
| KU-HAR dataset | Ankle (left) | 94 | 93 | 60 | 65 | 0.6006 |
|  | Ankle (right) | 65 | 84 | 69 | 94 | 0.4437 |
|  | Waist | 85 | 95 | 58 | 74 | 0.5629 |
|  | Back | 91 | 105 | 48 | 68 | 0.6107 |
|  | Head | 73 | 84 | 69 | 86 | 0.4850 |
|  | Wrist | 96 | 116 | 37 | 63 | **0.6575** |
|  | Mean |  |  |  |  | 0.5600 |
| SCDS/Ataxia dataset | Ankle (left) | 118 | 116 | 37 | 45 | 0.7421 |
|  | Ankle (right) | 105 | 91 | 62 | 58 | 0.6364 |
|  | Waist | 105 | 102 | 51 | 58 | 0.6583 |
|  | Back | 109 | 96 | 57 | 54 | 0.6626 |
|  | Head | 106 | 97 | 56 | 57 | 0.6523 |
|  | Wrist | 124 | 115 | 38 | 39 | **0.7631** |
|  | Mean |  |  |  |  | 0.6858 |

Supp Figure 2: Average kinematic signals in one gait cycle during the Walking with eyes open (<10s) task, for a healthy control (orange) and VS patient (blue), comparing head and wrist sensors.


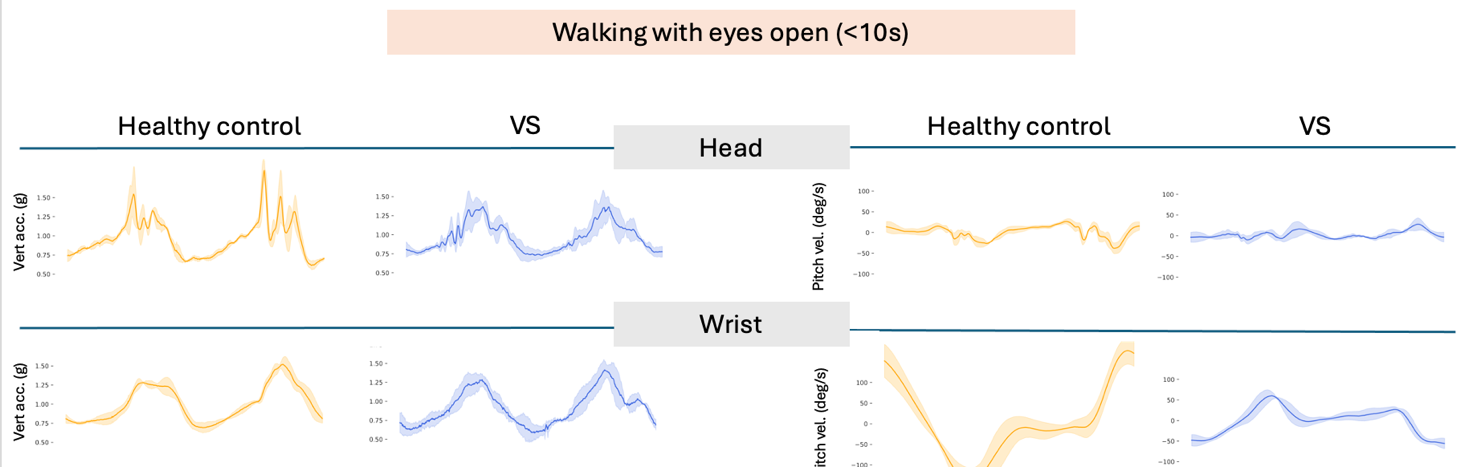


Supp Table 5: Confusion matrices and F1 scores for tasks from Table 8.

| **Task** | **% subjects per class (n)** | True positive | True negative | False positive | False negative | F1 score |  |
| --- | --- | --- | --- | --- | --- | --- | --- |
| Walking on level ground (*<*10s) | 25 (8) | 349 | 368 | 196 | 243 | 0.6139 |  |
|  | 50 (16) | 817 | 796 | 352 | 395 | 0.6863 |  |
|  | 75 (24) | 1224 | 1159 | 569 | 576 | 0.6813 |  |
|  | Baseline (32) | 1671 | 1569 | 726 | 714 | 0.6989 |  |
|  | Mean |  |  |  |  | 0.6701 |  |
| Walking with slow blinks (30s) | 25 (8) | 1714 | 1871 | 839 | 1053 | 0.6444 |  |
|  | 50 (16) | 3612 | 3619 | 1750 | 1874 | 0.6659 |  |
|  | 75 (24) | 5422 | 5836 | 2235 | 2717 | 0.6865 |  |
|  | Baseline (31) | 7317 | 7749 | 2601 | 3228 | 0.7151 |  |
|  | Mean |  |  |  |  | 0.678 |  |

Supp Table 6: Confusion matrices and F1 scores for tasks from Table 9.

| **Task** | **% samples per subject** | True positive | True negative | False positive | False negative | F1 score |  |
| --- | --- | --- | --- | --- | --- | --- | --- |
| Walking on level ground (*<*10s) | 25 | 342 | 444 | 156 | 243 | 0.6316 |  |
|  | 50 | 713 | 770 | 340 | 412 | 0.6547 |  |
|  | 75 | 1280 | 1085 | 610 | 520 | 0.6938 |  |
|  | Baseline (100) | 1671 | 1528 | 767 | 714 | 0.6929 |  |
|  | Mean |  |  |  |  | 0.6682 |  |
| Walking with slow blinks (30s) | 25 | 1769 | 1897 | 698 | 856 | 0.6948 |  |
|  | 50 | 3608 | 3828 | 1362 | 1672 | 0.704 |  |
|  | 75 | 5528 | 5642 | 2113 | 2392 | 0.7105 |  |
|  | Baseline (100) | 7330 | 7762 | 2588 | 3215 | 0.7164 |  |
|  | Mean |  |  |  |  | 0.7064 |  |

Supp Table 7: Standard parameters used for implementation of the machine learning model.

| **Activation function** | ReLU |
| --- | --- |
| **Output function** | Sigmoid, threshold at 0.5 |
| **Number of training epochs** | 100 |
| **Batch size (training, validation, testing)** | 64 |
| **Learning rate** | 0.1 |
| **Learning rate scheduler step size** | 25 |
| **Learning rate scheduler decay rate** | 0.1 |
| **Loss function** | Standard cross entropy |
| **Optimizer** | Adam |
| **Dropout rates** | 50% |
